# Abortion education in Canadian family medicine residency programs

**DOI:** 10.1186/s12909-018-1237-8

**Published:** 2018-06-01

**Authors:** Daniel T. Myran, Jillian Bardsley, Tania El Hindi, Kristine Whitehead

**Affiliations:** Department of Family Medicine, University of Ottawa, Ottawa, ON Canada

**Keywords:** Abortion training, Medical education, Family medicine residency, Medical abortion, Theory of planned behaviour

## Abstract

**Background:**

Abortion has been decriminalized in Canada since 1988 and is considered an essential medical service. There is concern that decreasing numbers of abortion providers may impair access to abortion. This study examined the quantity of exposure and education that Canadian family medicine residents receive on abortion during training and their preparation to provide abortions. In addition, the study assessed residents’ attitudes, intention and expressed competency to provide abortion in future practice and the association between medical training and changes in these factors.

**Methods:**

The authors developed a 21-item survey in consultation with experts in medical education. The survey was distributed online in 2016. A total of 1517 family medicine residents in their first, second and third year of training attending 8 English language schools across Canada were invited to participate. Associations between attitudes, education, exposure and intention were assessed using relative risks based on bivariate analysis of self-reported measures and odds ratios from ordered logistic regression.

**Results:**

The response rate was 28.7% (436/1517). The majority of residents, 79%, reported never observing or assisting with an abortion during training. Similarly, 80% of residents reported receiving less than 1 hour of formal education on abortion. Residents strongly supported receiving abortion education. Self reported exposure to a single abortion during training was associated with an increase in residents’ intention (RR = 1.95, 95% CI 1.54–2.47) and self-rated competency to provide a medical abortion (RR = 2.16, 95% CI 1.60–2.93). Twenty five percent of residents were unaware of ethical and legal requirements towards abortion provision and referral.

**Conclusions:**

Canadian family medicine residents receive little education or exposure to abortion during training most do not feel competent to provide abortion services. Residents expressed strong support for receiving abortion training. The Canadian College of Family Physicians curriculum does not currently include abortion as a training objective. The authors argue there is a need for family medicine training programs to increase education and exposure to abortion during residency, while respecting residents’ rights to opt out of such training. Failure to do so may impair future access to abortion provision.

**Electronic supplementary material:**

The online version of this article (10.1186/s12909-018-1237-8) contains supplementary material, which is available to authorized users.

## Background

Therapeutic abortion (TA), the intentional termination of a pregnancy, is a common procedure in Canada, with 28 abortions performed per 100 live births [1]. Approximately one in three Canadian women will have an abortion during her lifetime [2]. Abortion was decriminalized in 1988, and is fully covered under provincial and territorial health insurance as an essential health service [3]. However, women seeking access to abortion continue to face several challenges including: lack of trained providers and participating hospitals, regional disparities in access requiring long distance travel, inadequate provider and patient knowledge, and ongoing stigma towards abortion provision [3–5]. Similar trends of residual barriers to abortion access despite decriminalization or legalization have been observed across the developed world [6]. A consistent finding has been that low numbers of providers and lack of training opportunities significantly decrease the availability of abortion [6].

While there is no national information on the number and trends of abortion providers in Canada, data from British Columbia (BC) shows a general decline in the number of providers, with up to a 50% reduction in rural providers in the past two decades [7]. The geographic accessibility of abortion is related to the number of providers. In 2006, only 15.9% of Canadian hospitals offered abortion services, the majority of which were in urban areas [8] and until February 2017 the province of Prince Edward Island had no abortion providers [4]. A study of women seeking an abortion at a clinic in a major urban center in Canada found that more than 15% of women travelled between 101 and 1000 km to access an abortion provider. Indigenous, socioeconomically disadvantaged, and younger women were disproportionately impacted [5].

Exposure to abortion during medical education is likely associated with whether physicians provide abortion later in practice [9–11]. One study of practicing American obstetricians found that exposure to abortion during training strongly predicted whether obstetricians provided abortion during practice [9]. Two studies, one of Canadian obstetrics and gynecology residents, and one study of American family medicine residents, found that exposure to abortion during training was positively associated with residents’ expressed intention to provide abortions in future practice [10, 11]. However, Canadian family medicine and obstetrics residents may not be receiving exposure during training. A 2002 study found that the majority of Ontario family medicine residents and practicing physicians did not feel adequately trained to offer medical or surgical (procedural) abortions to their patients [12]. A recent study of Canadian Obstetrics and Gynecology residents found that 15% had received no training on abortion at all during residency, with an additional 34% reporting that abortion training occurred in their program only on a voluntary opt-in basis [13]. Similarly, two recent studies that surveyed medical school classes in Ontario and BC found that respondents had limited knowledge of abortion [14] and relatively few expressed intention to provide abortion in future practice [15].

Family doctors currently perform the majority of abortions in Canada, with the percentage of abortions provided by this group increasing over time [16]. In 2014–2015, family doctors provided 75.5% of the 86,824 reported TAs [16]. Consequently, the training and exposure that family medicine residents receive during residency may be of considerable importance to future access to abortion.

To our knowledge, this is the first study to examine national education, exposure, intention and self-expressed competency of Canadian family medicine residents with regards to abortion. We used the Theory of Planned Behaviour (TPB), a well-validated social psychology theory used in the prediction of future behaviour, to address the following objectives: [17, 18].

1. To examine the amount of education and exposure residents receive on abortion
2. To assess learner attitudes, intentions, and anticipated-competency around the provision of abortion care in their training and in practice
3. To examine the association between attitudes, perceived social norms, and perceived logistical difficulty on residents’ intention to provide abortion.
4. To examine the association between education and exposure on residents’ competency and intention towards abortion provision

## Methods

### Study setting

To answer our research questions, we designed a 21-item survey. Ethics approval was provided by the University of Ottawa and the University of Alberta Research Ethics Boards (REB). We approached the departments of family medicine at all English language family medicine residency programs in Canada that also had reciprocal REB agreements with the University of Alberta and the University of Ottawa for permission to distribute our survey. Universities that did not respond to invitations to participate or grant permission to distribute the survey were not included in the study. In total, the University of Ottawa, University of Toronto, Queen’s University, Western University, the Northern Ontario School of Medicine, University of Saskatchewan, University of Alberta, and University of British Columbia participated in the study. See Additional file 1: Appendix 1 for further details on exemption.

### Study population and sampling procedures

A total of 1517 family medicine residents currently enrolled in postgraduate year one, two and, three were eligible for study participation. Collectively the schools that participated enrolled 46.5% (1517 / 3265) of the country’s family medicine residents. The survey was administered online using the survey platform ‘Fluid Surveys™’. Participation was anonymous and voluntary, with survey completion implying consent. Eligible participants were recruited by email, in-class announcements and social media posts. University specific recruitment emails were sent on behalf of the researchers by the family medicine departments at each university, with the exception of the University of Toronto which posted an invitation to the survey in their monthly newsletter. Universities sent up to two follow up emails reminding residents to participate in the survey. Further details on study recruitment can be seen in Additional file 1: Appendix 1. Participants were entered into a draw to win one of three $20 gift certificates.

### Survey development and data collection

We used the Theory of Planned Behaviour (TPB) as a framework for survey design [17, 18]. The theory holds that intentions to engage in a behaviour are the strongest predictor of that future behaviour. Individual intention is best predicted by personal attitudes, how an individual perceives their community social norms, and the degree that a person feels they are able to perform the behaviour of interest. The TPB is accepted as being a strong predictor of future behaviour, including health and physician-related behavior [19, 20]. Questions from previous surveys examining abortion education were adapted with the aid of content experts in medical education and reproductive health to determine exposure to abortion in Canadian family medicine programs [9, 10, 15, 21]. The survey was piloted with five family medicine residents for feedback and administered between April 26th and June 26th, 2016.

#### Measures of interest

The main measures in our study were as follows (see Additional file 2: Appendix 2 for a copy of the survey with all measures including attitudes, social norms and perceived behavioral control):

- Education: Education was assessed with two questions: “In your residency so far, how many hours of teaching on abortion provision did you have in A) a formal academic setting (lecture, academic day presentation, online learning module etc) B) an *informal setting* (case discussions, bedside or in clinic teaching, informal presentations)”. Respondents were also presented with a short case with multiple choice responses to assess their knowledge of professional requirements.
- Exposure: Exposure to abortion was measured with the question “During your family medicine residency training have you assisted with or performed a medical or surgical abortion?” Residents were considered exposed if they answered yes.
- Intention to provide medical and surgical abortion: Intention was assessed using a 7-point Likert scale with the following questions “I intend to provide medical abortions in my future practice” and “I intend to provide *surgical* abortions in my future practice.”
- Self-reported competency: This measure was assessed using a 7-point Likert scale with the following three questions “By the end of residency, I expect to be competent to: A) Counsel women about abortion B) Perform medical abortions C) Perform at least one method of surgical abortions.

### Analysis of data

All analyses were conducted using SPSS Statistics version 24. An a priori decision, to simplify interpretation of the results, was made to divide intention responses into “intenders” and “non-intenders” with intenders answering 6 or 7 on the Likert scale and non-intenders answering 1–5 on the Likert scale when conducting bivariate analysis between exposure and intention and the components of the TRA and intention. We created a binary variable for exposure (yes/no), where residents who observed, assisted with or performed one or more medical or surgical abortions were considered exposed. We used Chi-square tests to examine the relationships between exposure to abortion and intention and self expressed competency. We calculated the Relative Risk (RR) based on the self-reported measures. We fit ordered logistic regressions to assess the association between education, exposure, and intention to provide medical abortions and included interaction terms to test for effect measure modification.

## Results

### Response rate and respondent characteristics

We received 436 responses for an overall response rate of 28.7%. Twenty-two participants were excluded for completing less than 50% of the survey, one individual who had indicated they attended a school which had not consented to participation was also excluded, with a final 413 (27.2%) responses retained for data analysis. See Additional file 1: Appendix 1 for the response rate by school. We report characteristics of our respondents in Table 1. The gender breakdown and mean age of participants in our study were comparable to the reported national averages for family medicine residents in 2014–2015 [22, 23].

**Table 1:** Respondent characteristics

	N (%)/Mean
Age	29.62 (SD 3.95)
Gender	
Male	123 (29.9%)
Female	289 70.1%)
Planned future practice size:	
Rural population 9999 or less	64 (15.5%)
Small town/city between 10,000 and 99,999)	138 (60.9%)
Urban population greater than 100,000	187 (23.6%)
Year of Training	
PGY-1	201 (48.6%)
PGY-2	202 (48.7%)
PGY-3	11 (2.7%)
Religious/moral objections to abortion	
Yes	73 (17.9%)
No	334 (82.1%)

### Education and exposure to abortion during residency

Fifty-seven percent of residents reported receiving no formal education on abortion and 80.2% received less than 1 hour during training. Twenty-seven percent of residents reported receiving no informal education on abortion and 64.2% received less than 1 hour. Twenty-two percent of residents reported receiving no education, formal or informal, during their training, and 45.7% reported receiving less than 1 hour.

**Fig. 1:**
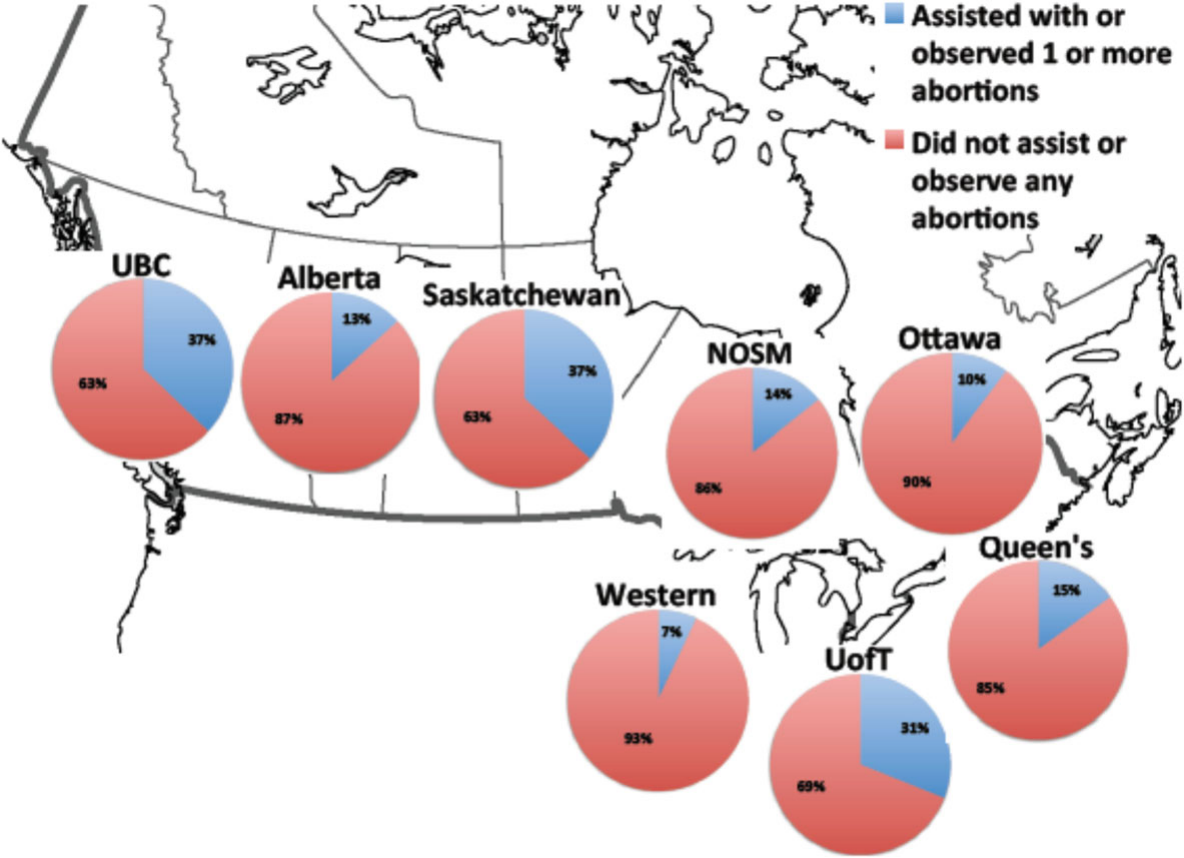
Exposure to abortion in residency training programs in Canada

We report the amount of exposure to abortion by university in Fig. 1. Twenty-one percent of residents reported being exposed to one or more abortions during residency. Of those exposed, 63.1% reported that their experiences occurred during a routine, scheduled rotation. The remainder reported that exposure to abortion occurred during an elective rotation.

### Attitudes towards abortion and medical education on abortion and intention to provide abortion

Sixty-one percent of residents in our survey very strongly or strongly supported receiving abortion training during residency. Table 2 shows a bivariate analysis of residents’ attitudes, social norms and perceived behavioural control, factors outlined in the TPB, stratified by intention to provide a medical abortion in future practice. Having positive attitudes, perceiving positive social norms, and anticipating relative ease of providing an abortion were all significantly associated with increased resident intention to provide medical abortion in future practice. When presented with a short case addressing professional requirements of abortion provision and referral, 25.6% of residents incorrectly responded that a physician was not required to refer to another provider if the physician was personally opposed to abortion provision.

**Table 2:** Mean scores of attitudes and demographics of residents stratified by intention to provide medical abortion

	Intender (148)^a^	Non-Intender (249)	
	Mean (SD)	Mean (SD)	
Positive attitudes towards abortion	6.39 (0.84)	4.28 (1.95)	*p* < 0.001
Positive perceived social norms towards abortion provision	4.92 (1.16)	3.00 (1.48)	*p* < 0.001
Anticipated ease of providing abortion	4.11 (1.52)	3.21 (1.46)	*p* < 0.001

^a^We defined positive intention as agree or strongly agree (6 or 7) on a 7 point Likert scale. There were 17 missing responses for intention

### Exposure to abortion during residency and intention and self-expressed competency to provide abortion

Exposure to abortion during residency was significantly associated with positive intentions to provide medical and surgical abortion, belief that abortion was within the scope of practice of family doctors and residents’ self-expressed competence to counsel on and provide abortions, see Table 3 and Table 4. Students who were exposed to abortion during a routine or elective rotation did not differ significantly in their intention to provide medical abortion (54.8% elective, vs 50% routine, Pearson chisquare = 0.1821 *p* = 0.670) or their anticipated competency to provide medical abortion (48.39% elective vs 46.15% routine, Pearsons chisquare = 0.0389 Pr = 0.844).

**Table 3:** Positive intention, scope of practice and competency by exposure to abortion during residency

	Exposed^^^ *N* = 88	Not Exposed *N* = 325	Relative Risk
^a^Intend to provide:			
Medical abortion in future practice	61.7% (50/81)	31.7% (97/306)	1.95 (1.54–2.47)
Surgical abortion in future practice	13.3% (11/83)	3.3% (10/304)	4.04 (1.78–9.22)
Within scope of practice for family doctor:			
Medical abortion	93% (80/86)	76.9% (240/312)	1.21 (1.11–1.32)
Surgical abortion	41.9% (36/86)	21.5% (67/312)	1.95 (1.40–2.70)
Competency to:			
Counsel on abortion	83.7% (72/86)	70.6% (221/313)	1.19 (1.05–1.33)
Provide a medical abortion	47.4% (41/86)	22% (69/313)	2.16 (1.60–2.93)
Provide a surgical abortion	12.8% (11/86)	5.8% (18/310)	2.22 (1.09–4.53)

^^^We defined exposure as being exposed to 1 or more abortions during residency

^a^We defined positive intention, within scope of practice, and competent, as agree or strongly agree (6 or 7) on a 7 point Likert scale

**Table 4:** Five ordinal logistic regression models fitting residents intention to provide medical abortion in future practice with the amount of formal and informal education received on abortion, exposure to abortion, gender, and interaction between informal education and exposure^a^

	Odds Ratio (95% CI)	*P*
Model 1. Formal education^b^	1.07 (0.86–1.34)	0.512
Model 2. Informal education^b^	1.22 (1.04–1.42)	0.014
Model 3. Exposed	3.58 (2.29–5.60)	< 0.0001
Model 4. Female	2.00 (1.36–2.94)	< 0.0001
Model 5. Fully adjusted model with formal and informal education, exposure, gender and interaction term.		
Exposed	5.52 (2.09–14.53)	0.001
Informal education	1.026 (0.81–1.28)	0.823
Female	1.84 (1.24–2.72)	0.002
Exposed^a^ * informal education	0.81 (0.54–1.21)	0.306

^a^Intention to provide medical abortion was measured on a 7-point likert scale with 7 being strongly intend to provide and 1 being strongly do not intend to provide

^b^Education was measured as zero hours, less than one hour, one to two hours, two to three hours, and more than three hours

### Education on abortion during residency and intention and self-expressed competency to provide abortion

We compared the self-reported competency of first and second year residents to provide a medical abortion by the end of residency. Residents in their second year of training (19.1%) were significantly less likely to report feeling competent to provide a medical abortion compared to first year residents (36%) (Pearsons chi-square 14.83 *p* = 0.001).

We fit ordinal logistic regressions to examine the association between formal education, informal education, gender, and exposure on intention to provide medical abortion by the end of residency (measured on a 7-point Likert scale). See Table 4 for model details. We found a trend towards increased intention to provide medical abortion at the end of residency with increased amount of formal education but no significant association. Each one-hour increase in informal education was associated with a 22% increase in the odds of expressing more positive intentions to provide medical abortion (*p* = 0.014).

## Discussion

This study found that Canadian family medicine residents receive little education on, or exposure to abortion during their family medicine training. However, respondents to our survey generally held positive attitudes towards the provision of abortion and supported the inclusion of abortion training in their post-graduate programs. Residents who received formal education on abortion showed a non-significant trend towards increased intention to provide medical abortion in future practice, while increased informal education was significantly associated with increased intention to provide medical abortion. Residents who reported exposure to abortion during training were more likely to intend to provide medical abortions, believe that abortion should be part of postgraduate training and believe that abortion provision was within the scope of practice for family physicians.

Our data on limited education and exposure are consistent with a 2002 study of family medicine residents in Thunder Bay and Hamilton, Ontario, which found that only 39.1% of residents reported education on abortion during residency [12]. The lack of abortion education during residency may be partially explained by its absence from the College of Family Physicians of Canada (CFPC) list of 99 priority topics and the Medical Council of Canada (MCC) objectives for licensure [24, 25]. While we find the low levels of education and exposure to abortion concerning, we are further alarmed by the finding that one-quarter of respondents in our survey were unaware of ethical and legal requirements towards abortion referral for non-providers. This finding suggests that a substantial portion of graduating family medicine residents may not provide the legally required standard of care.

The positive association between exposure to abortion during training and self-expressed competency and intention to provide abortion is consistent with the literature. Previous studies of residents found that the number of manual vacuum aspirations performed in residency was positively correlated with intention to provide abortions in future practice [11]. The association between higher self-reported competency and intention to provide abortion for residents did not differ for residents who were exposed to abortion on an elective or on a regularly scheduled rotation. This comparison controls for selection bias, where individuals voluntarily exposed to abortion may differ from individuals who did not volunteer, and suggests that exposure may result in increased intention and competency in abortion provision, regardless of initial interest.

The absence of a significant association between residents’ formal education and intention to provide abortion was unexpected and merits further investigation. One speculation is the majority of the limited formal education that residents receive may focus on ethical considerations of abortion rather than actual details of abortion provision. Prior work examining education on abortion in the medical school curriculum has found a disproportionate focus on ethics over clinical knowledge [26]. Further research is needed to examine the type of education on abortion that residents are receiving and how to better deliver impactful education on this topic.

Canadian women face several barriers to receiving abortion care. The absence of trained providers in non-urban communities limits access to this essential health service (3,4,5). Given that family physicians provide approximately three in four therapeutic abortions in Canada and that the number of Canadian providers may be declining [3, 7, 8] there is a need to train new family physicians to provide abortions. It is hoped that the recent Health Canada approval of mifepristone will streamline the provision of medical terminations thus improving access to abortion [7, 27]. However, studies examining the introduction of mifepristone in the United States found that although rates of medical abortion increase, overall access did not improve [28, 29]. While there are substantial cultural, funding, and health systems differences between Canada and the United States with regards to abortion, lack of education for providers appears to be a shared factor. In a study of New Mexico physicians, lack of training in medical abortion was the most commonly reported barrier to abortion provision [29]. We are similarly concerned that the current lack of resident education on abortion could limit potential improvements to abortion access in Canada from mifepristone.

Implementing abortion training in academic family medicine units is possible. Two studies have described the successful integration of abortion provision at Beth Israel Residency Program in New York, and at the University of New Mexico (UNM) despite facing cultural, logistic, financial and political barriers. At UNM the authors credited the following elements for the program success; adding mifepristone to the hospital formulary, normalizing manual vacuum aspiration by first introducing it for use in incomplete and missed spontaneous abortions, holding a values workshops emphasizing and patient-centred care, and reassurance that staff with moral objections were not obligated to participate [30].

The results of the positive association between attitudes, perceived social norms and anticipated ease of provision with intention to provide medical abortion is consistent with predictions of the TPB. These results suggest that efforts to promote positive social norms and reduce perceived logistic difficulties of abortion provision would result in increases intention by residents to provide abortion. We argue that departments of family medicine could create positive social norms, decrease stigma associated with abortion provision, and increase abortion related competency, by creating opportunities for routine clinical exposure to abortion for all residents. Evidence from programs that have integrated abortion provision into residency training have found a shift towards more positive social norms and attitudes regarding abortion [30, 31]. Academic programs would be more likely to educate and expose residents to abortion if the Canadian College of Family Medicine added abortion to their list of “99 priority topics” as these topics form the backbone of the competency based curriculum in family medicine residency. Further studies would be needed to see if education and exposure interventions increased the actual number of abortion providers in Canada.

### Limitations

Our study has several limitations including the cross-sectional design, low overall response rate, and self-report outcomes. While we found positive associations between variables such as exposure and intentions to provide abortion as well as expressed competency to do so, we are unable to establish causal relationships given the cross-sectional nature of the study. Future studies could follow family medicine residents longitudinally through training to better examine the impact of education and exposure on abortion related measures.

The overall response rate of 28.7% and the lack of data from French language and Maritime programs were limitations to our study. Despite the low response rate, we believe that several factors support the validity of our findings. First, overall trends did not differ from those observed at the University of Ottawa, which had over a 60% response rate, and other programs. Second, measures such as reported education and exposure reflect objective aspects of residency programs and are thus less likely to be impacted by response bias. Third, our respondents’ rate of religious or moral objections to abortion was consistent with previously reported rates suggesting a representative sample [11, 14].

Finally, all measures in the survey, including competency which can be measured objectively, were self-reported. As residents may not be good judges or their own competency, and could be subject to social desirability bias, further research could focus on objective measurements of competency for abortion provision.

## Conclusions

Despite residents holding strongly positive views on abortion family medicine training programs in Canada provide little education and exposure to abortion. The majority of family medicine residents do not feel competent to provide abortion services. These findings are concerning given studies highlighting existing difficulties accessing abortion services in Canada. Multiple examples of successful integration of abortion training into family medicine residency have been documented in the United States. We argue that there is an urgent need for family medicine programs across the country to develop and integrate education and clinical exposure on abortion provision into training. Medical education programs should focus on normalizing abortion provision through routine clinical exposure for family medicine residents, while respecting individual residents’ rights to opt out of training.

## Additional files

Additional file 1: **Appendix 1** contains detailed information about school participation, the recruitment strategy at individual universities, and the response rate for individual universities. (DOCX 15 kb)

Additional file 2: **Appendix 2** contains a copy of the survey questions and the order that they were presented in. (DOCX 18 kb)

## Abbreviations

BC: British Columbia
CFPC: College of Family Physicians of Canada
MCC: Council of Canada
REB: Research Ethics Board
TA: Therapeutic Abortion
TPB: Theory of Planned Behaviour
