# Supplementary material for: Abortion education in Canadian family medicine residency programs
Source: BMC Med Educ. 2018 Jun 1;18:121. doi: 10.1186/s12909-018-1237-8 (PMC5984743; doi:10.1186/s12909-018-1237-8)
Supplement: Supplementary file 1 — Appendix 1 contains detailed information about school participation, the recruitment strategy at individual universities, and the response rate for individual universities. (DOCX 15 kb) [file 12909_2018_1237_MOESM1_ESM.docx]

**Additional file 1 Appendix 1. Exemptions from Participation, Response Rate, and Recruitment by University**

*Exemptions from Participation*

McGill, Memorial University of Newfoundland, Dalhousie, and the Northern Ontario School of Medicine (NOSM) - Thunder Bay campus, did not have either a Research and Ethics Board (REB) co-operation with Ottawa or an expedited REB process, and hence were excluded from invitation to participate. The University of Manitoba did not respond to requests to participate. Permission to distribute the survey to residents was requested but not granted by the department of family medicine at McMaster

*Recruitment by University*

The University of Toronto distributed one invitation to participate in a departmental newsletter and no follow-up invitations were issued. UBC sent only one follow-up email. An in-class announcement was made at the Universities of Ottawa and Alberta. One posting on the social media platform Facebook was made for the classes at Ottawa, Queen’s, Western and Alberta.

*Response rate by school*

Our response rate by school was as follows and is summarized in Table 1; University of Ottawa 58% (87/150), NOSM – Laurentian Campus 37.8% (14/37), Queen’s University 34.7% (60/173), University of Toronto 12.9% (45/350), Western University 24.3% (44/181), University of Saskatchewan 35.8% (38/106), University of Alberta 22.4% (38/170), University of British Columbia 24.6% (436 / 350).

Table 1. Response rate by residency program

| **University** | **Respondents/Total Enrolled** | **Response Rate** |
| --- | --- | --- |
| University of Ottawa | 87/150 | 58% |
| NOSM – Laurentian Campus | 14/37 | 37.8% |
| Queen’s University | 60/173 | 34.7% |
| University of Toronto | 45/350 | 12.9% |
| Western University* | 44/181 | 24.3% |
| University of Saskatchewan | 38/106 | 35.8% |
| University of Alberta | 38/170 | 22.4% |
| University of British Columbia | 86/350 | 24.6% |
| Total | 436/1517 | 28.7% |

*All information on number of enrolled residents was provided by the programs with the exception of Western University which did not supply this information. The number of enrolled residents at Western University was taken from <http://www.caper.ca/~assets/documents/pdf_2014-15_CAPER_Census.pdf>
