# Supplementary material for: Abortion education in Canadian family medicine residency programs
Source: BMC Med Educ. 2018 Jun 1;18:121. doi: 10.1186/s12909-018-1237-8 (PMC5984743; doi:10.1186/s12909-018-1237-8)
Supplement: Supplementary file 2 — Appendix 2 contains a copy of the survey questions and the order that they were presented in. (DOCX 18 kb) [file 12909_2018_1237_MOESM2_ESM.docx]

**Additional file 2 Appendix 2. Copy of Survey**

**Demographic Questions**

**1.  What is your age?**

____

**2.  What is your gender?**

| ¨    a) Male  ¨    b) Female  ¨    c) Other |
| --- |

**3.  What year of training are you in?**

a) PGY1

b) PGY 2

c) PGY 3

**4.  What is the population size of the area that you plan to establish your medical practice in?**

a) Remote/isolated (population <1, 000)

b) Rural (population between 1,000 and 9,999)

c) Small town/city (population between 10,000 and 49,999)

d) Medium town/city (population between 50,000 and 99,999)

e) Large town/city (population between 100,000 and 1,000,000)

f) Major urban centre (population >1,000,000)

g) Unsure

**5.  What university is your residency program associated with?**

1. University of British Columbia
2. University of Alberta
3. University of Calgary
4. University of Saskatchewan
5. University of Western Ontario
6. University of Toronto
7. Queen’s University
8. Northern Ontario School of Medicine- Laurentian affiliated
9. University of Ottawa
10. Other (please fill in)

**6. In your residency so far, how many hours of teaching on abortion provision did you have in**

- a *formal academic setting* (lecture, academic day presentation, online learning module etc).

a) 0

b)<1

c)1-2

d) >2-3

3) >3

- an *informal setting* (case discussions, bedside or in clinic teaching, informal presentations).

a) 0

b)<1

c)1-2

d) >2-3

3) >3

**7. During your family medicine residency training have you assisted with or performed a medical or surgical abortion?**

a) Yes

b) No

***In a pop-up box if yes to question 7. If no program skips to question 12.***

**If yes this was part of**

a) A routine or opt-out rotation, i.e. a scheduled block of time within residency training with the option to not participate

b) An elective or opt-in rotation, i.e. not a scheduled block of time but available to interested residents during elective rotations

c) Not available at all; interested residents trained in free time, outside of scheduled academic training

d) Other (please describe)

If yes

**8. How difficult was this training to arrange?**

1. Very difficult
2. Difficult
3. Somewhat Difficult
4. Neither difficult nor easy
5. Somewhat Easy
6. Easy
7. Very Easy

If yes…

**9. Please indicate your agreement with the following statement:**

|  | 1  Strongly Disagree | 2 Disagree | 3  Somewhat disagree | 4  Neither disagree nor agree | 5  Somewhat agree | 6  Agree | 7  Strongly Agree |
| --- | --- | --- | --- | --- | --- | --- | --- |
| In general, the medical faculty and administrative staff of my family medicine program are supportive of residents receiving training in abortion provision. |  |  |  |  |  |  |  |

In a pop-up box if yes to 7:

**10. How many medical abortions did you observe, assist with or perform thus far during residency?**

____

**11. How many surgical abortions did you observe, assist with or perform thus far during residency?**

____

**12. Please indicate your agreement with each of the following statements: By the end of residency, I expect to be competent to:**

|  | 1  Strongly Disagree | 2  Disagree | 3  Somewhat disagree | 4  Neither disagree nor agree | 5  Somewhat agree | 6  Agree | 7  Strongly Agree |
| --- | --- | --- | --- | --- | --- | --- | --- |
| Counsel women about abortion |  |  |  |  |  |  |  |
| Perform medical abortions |  |  |  |  |  |  |  |
| Perform at least one method of surgical abortions (vacuum aspiration, dilation and curettage) |  |  |  |  |  |  |  |

**13. Do you have religious or moral objections to elective abortion?**

a) Yes

b) No

**14. Please indicate your agreement with each of the following statements:**

|  | Yes | No |
| --- | --- | --- |
| Medical abortion is part of the scope of practice for Canadian family doctors. |  |  |
| Surgical abortion is part of the scope of practice for Canadian family doctors. |  |  |

**15.**

| For me, performing a medical abortion during future practice would be: | Bad | 1 | 2 | 3 | 4 | 5 | 6 | 7 | Good |
| --- | --- | --- | --- | --- | --- | --- | --- | --- | --- |
|  | Harmful | 1 | 2 | 3 | 4 | 5 | 6 | 7 | Beneficial |
|  | Wrong | 1 | 2 | 3 | 4 | 5 | 6 | 7 | Right |

**16. Please indicate your agreement with each of the following statements:**

|  | 1  Strongly Disagree | 2 Disagree | 3  Somewhat disagree | 4  Neither disagree nor agree | 5  Somewhat agree | 6  Agree | 7  Strongly Agree |
| --- | --- | --- | --- | --- | --- | --- | --- |
| Providing medical abortion as part of my future practice would be logistically difficult (finding support staff, obtaining supplies and medications, security, etc.). |  |  |  |  |  |  |  |
| I intend to provide *medical* abortions in my future practice. |  |  |  |  |  |  |  |
| I intend to provide *surgical* abortions in my future practice. |  |  |  |  |  |  |  |
| Most people who are important to me think that I should provide medical abortions during my future practice. |  |  |  |  |  |  |  |

**17. Abortion provision should be part of my Family Medicine training.**

| 1  Strongly Disagree | 2 Disagree | 3 Somewhat disagree | 4  Neither disagree nor agree | 5  Somewhat agree | 6  Agree | 7  Strongly Agree |
| --- | --- | --- | --- | --- | --- | --- |

Multiple Choice Questions

A * represent the correct answer

**1. What is the most effective method of medical abortion?**

a) Misoprostol 800 mcg buccal or vaginal

*b) Methotrexate 50 mg /m^2^ oral or IM and Misoprostol 800 mcg buccal or vaginal

*c) Mifepristone 200 mg oral and misoprostol 800 mcg buccal or vaginal

d) Mifepristone 200 mg oral

e) Mifepristone 200 mg oral and methotrexate 50 mg/m^2^ oral or IM

*Methotrexate and misoprostol or mifepristone and misoprostol were both accepted as correct methods of medical abortion.

**2. What is the recommended gestational limit for a medical abortion, as per the Society of Obstetricians and Gynecologists Canada (SOGC)?**

a) Up to 6 weeks

*b) Up to 8 weeks

c) Up to 10 weeks

d) Up to 12 weeks

**3) A 34 year-old presents to an urban practice requesting an abortion. If the physician at the clinic objects to abortion on moral grounds what is their minimum professional obligation to the patient?**

a) refer her to a pregnancy crisis center, if available, for options counseling

b) inform her about their moral objections and end the appointment

c) inform her that abortion services are by self-referral and she should look up a provider

*d) provide the contact information for a clinic or physician who can provide an abortion

e) perform the abortion yourself despite their objections

**4)   Compared to carrying a pregnancy to term and delivery, abortion is more likely to result in? Select all that apply:**

a)      Infertility

b)      Infection

c)      Depression

d) Breast Cancer

*e)      None of the above
